# 

**Published:** 1890-09

**Authors:** 


					Ir=
SEPTEMBER, 1890.
Vol. VIII. No. 29.
THE
BRISTOL
MEDICO-CHIRURGICAL
JOURNAL
&
\ &
\ K
V
\ j Lf'"
\V /#
V-: On.
\ i. c? ?
a <&uarterlg Journal of tbe jfflbeMcal Sciences for tbe West
of BnglanD and Soutb HClales
PUBLISHED UNDER THE AUSPICES OF
THE BRISTOL MEDICO-CHIRURGICAL SOCIETY".
editor :
J. GREIG SMITH, M.A., F.R.S.E.
BRISTOL: J. W. ARROW SMITH ;
London: J. & A. Churchill, 11 New Burlington Street.
Price One Shilling and Sixpence.
?

				

